# Motor-Based Interventions in Children with Developmental Coordination Disorder: A Systematic Review and Meta-analysis of Randomised Controlled Trials

**DOI:** 10.1186/s40798-025-00833-w

**Published:** 2025-05-26

**Authors:** Jiaxin Gao, Yihan Yang, Xiaqing Xu, Dunbing Huang, Yangxin Wu, Hongfei Ren, Anren Zhang, Xiaohua Ke, Wei Song

**Affiliations:** 1 School of Health and Rehabilitation, Chengdu University of Traditional Chinese Medicine, Chengdu, China; 2 Department of Rehabilitation Medicine, Shanghai Fourth People’s Hospital, School of Medicine, Tongji University, Shanghai, China; 3 College of Rehabilitation Medicine, Fujian University of Traditional Chinese Medicine, Fuzhou, China

## Abstract

**Background:**

Developmental coordination disorder (DCD) is a neuromotor disorder in children that is characterized by significant difficulties in fine and gross motor skills. The main interventions for children with DCD are motor-based interventions (MBI), and a large number of relevant randomized controlled trials (RCTs) have emerged in recent years, but the efficacy of different types of MBI on different outcome parameters is unclear. Therefore, the aim of this study was to assess the effectiveness of MBI on standardized motor tests, body functions, activity and participation performance, and psychosocial factors in children with DCD, and to explore the differential effects of different types of interventions (including process-oriented, task-oriented, or combined task- and process-oriented) on the above outcome parameters.

**Methods:**

We conducted a systematic literature search of all studies published in PubMed, Web of Science, Embase, and the Cochrane Library up to January 31, 2024 to compile all RCTs on MBI for children with DCD. Participants in this study were children with DCD between the ages of 3 and 17, using standardised motor skills tests, body function, activity and participation performance, or psychosocial factors as study outcomes. We assessed the risk of bias for each study and the overall risk of bias using Cochrane's ‘risk of bias’ tool. Quantitative syntheses (meta-analyses) were conducted with effect sizes expressed as Hedges' g.

**Results:**

A total of 32 studies were included in the meta-analysis. The results showed that MBI significantly improved the overall motor skills (g = 1.00, 95%CI [0.48,1.52], p < 0.001), balance function (g = 0.57, 95%CI [0.17,0.97], p = 0.005), cognitive function (g = 1.53, 95%CI [0.67,2.39], p = 0.001), muscle function (g = 0.91, 95%CI [0.17,1.66], p = 0.017), coordination function (g = 0.47, 95%CI [0.04,0.90], p = 0.032), visual function (g = 0.61, 95%CI [0.15,1.08], p = 0.009), sensory function (g = 0.85, 95%CI [0.34,1.35], p = 0.001), sensory organization function (g = 0.61, 95%CI [0.27,0.96], p = 0.001) and activity performance (g = 0.71, 95%CI [0.23,1.19], p = 0.004), but improvements in children's psychosocial factors (g = 0.71, 95%CI [− 0.08,1.50], p = 0.079) were not significant, nor were improvements in children's participation levels observed with MBI. Subgroup analyses further revealed that task-oriented training significantly improved overall motor skills, balance function, and activity performance in children with DCD, and that combined task- and process-oriented training also significantly improved overall motor skills in children with DCD.

**Conclusions:**

MBI demonstrates significant positive effects on enhancing standardized motor test scores, body functions, and levels of activity performance in children with DCD. However, no improvements were observed in children's participation levels, and no statistically significant effects were observed on psychosocial outcomes. Our findings further highlight the comparative effectiveness of intervention strategies. Task-oriented approaches significantly improved overall motor skills, balance, cognitive function, and activity performance, while combined task- and process-oriented strategies also enhanced overall motor skills. In contrast, given the limited number of included studies, the effects of process-oriented strategies on motor skills and activity performance, as well as the impact of combined strategies on activity performance, remain inconclusive. In conclusion, our comprehensive study suggests the preference for employing task-oriented strategies or training underlying processes within task-oriented training for children with DCD.

**Registration:**

The protocol of the investigation was registered in PROSPERO (ID: CRD42024499574).

**Key Points:**

- Motor-based interventions significantly improved standardized motor test scores, body functions, and activity performance levels in children with DCD, but no improvements were observed in participation levels or psychosocial outcomes.
- Task-oriented strategies were highly effective in improving motor skills, balance, cognitive function, and activity performance, while combined task- and process-oriented approaches also enhanced overall motor skills.
- The effects of process-oriented approaches on motor skills and activity performance, as well as the impact of combined strategies on activity performance, remain inconclusive due to the limited number of studies, emphasizing the need for further research.

**Supplementary Information:**

The online version contains supplementary material available at 10.1186/s40798-025-00833-w.

## Background

Developmental Coordination Disorder (DCD) is a common neurodevelopmental disorder in children [1] with an estimated prevalence of 2–20% [2]. Children with DCD exhibit a broad spectrum of coordinated motor skill difficulties, encompassing both gross and fine motor skills. This is evidenced by impaired postural control, balance, and significant deficits in sensory-motor coordination [3]. Importantly, these motor deficits do not stem from physical, intellectual, or neurological abnormalities [3]. Research indicates that children with DCD experience deficits across various aspects of motor control, including movement planning and anticipatory control, as well as fundamental processes of motor learning and cognitive control [2]. The impact of DCD extends beyond motor and cognitive functions, affecting the ability to engage in daily activities and social participation [4–6]. Due to motor skill deficits, children experience challenges in carrying out activities essential for daily living, including self-care, self-maintenance, and mobility [2]. Additionally, they encounter significant difficulties in tasks such as writing, drawing, and participating in ball games [7, 8]. These difficulties substantially impact their academic performance and overall mobility. Furthermore, children with DCD often exhibit a variety of avoidance behaviors and experience psychosocial effects [9]. Specifically, they tend to have markedly reduced self-esteem regarding their physical abilities, and their self-concept and self-efficacy are frequently lower as well [10–13]. Peer rejection stemming from motor difficulties can lead to varying degrees of social deficits in children with DCD [14]. Moreover, they are more prone to being targeted for bullying [15, 16]. This can result in significant emotional challenges, such as depression and anxiety, which can detrimentally impact a child's mental health [17]. According to the International classification of functioning, disability and health (ICF) [18], DCD influences all aspects of children's body functions and structures, activities and participation. Hence, early intervention is imperative to enhance motor skills, physical and mental well-being, and social engagement among children with DCD [5, 19].

At present, interventions for children with DCD encompass motor-based interventions (MBI), pharmacological treatments, and psychosocial interventions specifically designed to tackle the diverse range of psychosocial challenges associated with DCD [2, 9, 20]. Of these, MBI is the most prominent intervention strategy for children with DCD, and MBI also can positively impact children's motor skills, and as children's motor abilities improve, their self-concept and self-efficacy also tend to enhance. This, in turn, promotes positive self-evaluation and effectively addresses various psychosocial issues often associated with DCD [21]. MBI typically fall into two main categories [19]: one being the bottom-up process-oriented approach, which attributes impaired motor skills in children with disabilities to impairment in specific body functions or structures, particularly neural structures, or sensory processes like vision or proprioception [3]. This approach aims to enhance various body functions such as perception, sensory integration, balance, and muscle strength through interventions oriented towards body function. Specific strategies include sensory integration training, kinesthetic training, and strength training. The other category involves top-down task-oriented approaches, such as neuromotor task training, cognitively oriented occupational performance, and motor imagery training. These approaches prioritize the improvement of mastery and performance in specific tasks, emphasizing the sustained interaction between the activity, the child, and the environment. The primary goal is to enhance performance in specific activities or increase participation, thus falling under activity or participation-oriented interventions [9]. Previous studies [19, 22, 23] highlighted that the impairments associated with DCD are multifaceted, and therefore recommended using a multi-level approach that incorporates both task-oriented and process-oriented strategies (i.e., a combined process- and task-oriented approach). This approach involved the flexible application of multi-level therapeutic theories to design effective intervention strategies. Such strategies were intended to accommodate individual differences in performance, learning styles, and progress, ultimately improving functional impairments in children with DCD.

The positive effects of process-oriented and activity-oriented MBI for children with DCD were validated in a 2018 meta-analysis. However, the evidence from this study relied on data obtained from a single group (intervention group) of trials pre- and post- the intervention data [5], lacking high-quality evidence of effects from randomized controlled trials (RCTs). In recent years, a significant number of RCTs have investigated the impacts of MBI on motor skills, body functions and structures, activities and participation, as well as psychosocial aspects in children with DCD. However, the effects of various types of MBI on different outcome indicators vary. As a result, parents and healthcare providers of children with DCD require a more comprehensive review to select the most suitable intervention for their children. Systematic integration of these RCTs is essential to further bolster the level of evidence supporting the positive efficacy of MBI in DCD. Additionally, it is crucial to analyze different types of MBIs to identify their varying intervention effects. Therefore, the objectives of this systematic review and meta-analysis were as follows: (1) to evaluate the intervention effects of MBI on children with DCD using data from RCTs; (2) to assess the effects of different types of MBI (including process-oriented, task-oriented, or combined task- and process- oriented) on the overall motor skills, body structures and functions, and activities and participation of children with DCD; (3) to evaluate the intervention effects of MBI on the psychosocial aspects of children with DCD. In this review, a comprehensive overview of MBI in children with DCD will be presented, aiming to offer evidence-based recommendations for the development of exercise-based interventions tailored to this population.

## Method

### Registration of the Systematic Review Protocol

The protocol of the investigation was registered in the International Prospective Register of Systematic Reviews PROSPERO (ID: CRD42024499574). This systematic review and meta-analysis were conducted in accordance with the Preferred Reporting Items for Systematic Reviews and Meta-Analyses (PRISMA) [24].

### Search Strategy

Four individuals (JXG, XXQ, HFR, YXW) conducted a systematic literature search for all published studies from database creation to January 31, 2024 in PubMed, Web of Science, Embase and the Cochrane Library. Online Resource 1 summarises the terms used.

### Criteria for Selecting Meta-analyses

#### Inclusion Criteria

*Types of Studies*

RCTs.

*Types of Participants*

Participants were children aged 3–17 years with DCD, including those with probable DCD (pDCD). Participants were either diagnosed with DCD using diagnostic criteria from the Diagnostic and Statistical Manual of Mental Disorders, Fourth Edition (DSM-IV) [25]/fifth edition (DSM-V) [26], and International Classification of Diseases(ICD) [27], or assessed for motor impairments (i.e., p-DCD) using standardized motor tests, such as the Movement Assessment Battery for Children Test (MABC)-1/2 [28, 29] or the Bruininks-Oseretsky Test of Motor Proficiency (BOTMP) [30].

*Types of Interventions*

The intervention group was any intervention based on motor training, while the control group was passive control.

*Types of Outcome Measures*

Primary outcome indicators: Standardised Movement Skills Tests: the MABC-1 or − 2, the BOTMP-1 or − 2, the Test of Gross Motor Development (TGMD).

Secondary outcome indicators: (1) ICF-based classification of body functions (e.g., balance function, cognitive function (inhibitory control, attention network, visuospatial working memory, action planning), muscle function, etc.) and activities (e.g., academic level, occupational performance) and participation (e.g. participation in daily living activities (Questionnaire on Parent’s Perception of Changes in their Child’s Participation) etc.). (2) Measures of psychosocial factors (self-esteem, self concept) such as the Pictorial Scale for Perceived Competence and Social Acceptance for Young Children (PSPCSA), Spence’s Child Anxiety Scale (SCAS), Self-perceived physical competence (SPC); Tennessee Self-Concept Scale-Child Form (TSCS-CF); Child Anxiety Scale (CAS), etc. The outcome measurement tools include subjective questionnaires reported by parents, teachers, or the children themselves. For studies reporting multiple outcomes, we prioritized those most relevant to the main research question and those with significant implications for clinical practice or real-world applications, these outcomes best reflect treatment decisions or health outcomes for patients.

The division in standardized motor skills tests and the ICF classification is not mutually exclusive. Standardized motor skill tests provide a holistic evaluation of children's overall motor abilities, assessing their motor coordination skills in both body functions and activities. In contrast, the outcomes within the ICF classification further refine these motor abilities across the levels of body function, activity, and participation.

#### Exclusion Criteria

Studies that met any of the following criteria were excluded: (1) The diagnostic criteria used were not clearly reported; (2) No randomized controlled trail; (3) Published in languages other than English.

### Data Extraction

All retrieved documents were first imported into EndNote X9 and duplicates were removed using this software. Two reviewers (JXG, XQX) independently screened the titles and abstracts of the literature one after the other, eliminating obviously irrelevant records. The full text of the remaining records was then retrieved and final inclusion was determined. We recorded the selection process and summarized the information in a PRISMA flow diagram [31]. Two independent reviewers (HFR, YXW) extracted data from the included studies on (1) study characteristics (year of publication; geographic region); (2) sample size and patient characteristics (age; diagnostic criteria; co-morbidity); (3) intervention parameters (intervention-each intervention was broadly categorized according to process-oriented (i.e. the training performed was designed to improve the target body functions that are considered to underlie the reported functional motor problem, e.g. strength training, neuromuscular training, etc.), task-oriented (the task was designed to improve the child's performance of the activity, e.g. task-specific methods, CO-OP, video games, etc.) and combined task- and process-oriented (a combination of process oriented and task oriented) approaches; duration of treatment; frequency); (4) outcome measures; (5) results. Extracted data were cross-reviewed by two reviewers (JXG, YHY), and disagreements, if any, were discussed and resolved with the other author (XQX). For quantitative analysis, the mean difference as well as the standard deviation (SD) of the outcomes associated with the intervention and control groups have been collected. In case of missing data, we contacted the original author.

### Study Quality Assessment

Two review authors (JXG, YHY) independently assessed the risk of bias for each study and the overall risk of bias by using Cochrane's 'risk of bias' tool [32]. We assessed the risk of bias for each included study against the following key criteria: I random sequence generation; II allocation concealment; III blinding of participants and personnel; IV blinding of outcome assessment; V incomplete outcome data; VI selective reporting; VII other bias. Each of the above areas was clearly judged to be at low risk of bias, high risk of bias, or unclear risk of bias.

### Statistical Analysis

Comprehensive meta-analysis (version 3.0, Biostat Inc, Englewood, NJ, USA) was used for meta-analysis to calculate individual study and pooled effect sizes (ES). ES were expressed as Hedges' g. They were calculated by dividing the difference between the means of the treatment and control groups by the standard deviation and weighted according to sample size to correct for small-sample bias [33]. A random effects model was used for all calculations. In addition, 95% confidence intervals for effect sizes were reported. Standardized mean differences (g) less than 0.2, 0.5 and greater than 0.8 were considered small, medium and large effects, respectively [34]. Between-study heterogeneity was assessed using the Q-test and quantified using the I^2^ statistic. I^2^ quantifies the degree of heterogeneity by providing the percentage of variance attributable to between-study variation [32]. 0% denotes no heterogeneity, 25%, 50%, and 75% indicate low, medium, and high heterogeneity, respectively.

For the subgroup analyses, we analyzed the primary and secondary outcome indicators in groups of differently oriented interventions, specifically process-oriented, task-oriented, or combined task- and process-oriented. Moreover, The MABC test includes three age groups (3–6 years (1); 7–10 years (2); 11–16 years (3)) and three major categories (gross motor skills, fine motor skills, and hand–eye coordination skills). The specific test items are as follows: Fine Motor Skills (Manual dexterity): (1) Posting coins(i)/Placing pegs(ii)/Turning pegs(iii). (2) Threading beads(i)/Threading lace(ii)/Triangle with nuts and bolts(iii). (3) Drawing Trail(i&iii&iii); Hand–eye coordination skills (Aiming&catching): (1) Catching beanbag(i)/Catching with two hands(ii)/Catching with one hands(iii). (2) Throwing beandag onto mat(i&ii)/Throwing at wall target(iii); Gross Motor Skills (Balance): (1) One-leg balance(i)/One-board balance(ii)/Two-board balance(iii). (2) Walking heels raised(i)/Walking toe-to-heel forwards(ii)/Walking toe-to-heel backwards(iii). (3) Jumping on mats(i)/hopping on mats(ii)/Zig-zag hopping(iii). The three major categories in the MABC test comprehensively reflect the coordinated motor skills developed during childhood. Therefore, we conducted a subgroup analysis based on these three main abilities: Gross Motor Skills, Fine Motor Skills, and Hand–eye Coordination Skills.

The certainty of the evidence for each outcome was assessed by using the Grading of Recommendations Assessment, Development and Evaluation criteria (GRADE) [35] and approach to conduct management recommendations by the GRADEpro Guideline Development Tool (GDT) online (https://gradepro.org/), with the certainty of evidence categorised as high, moderate, low and very low.

## Results

### Study Selection

**Fig. 1:**
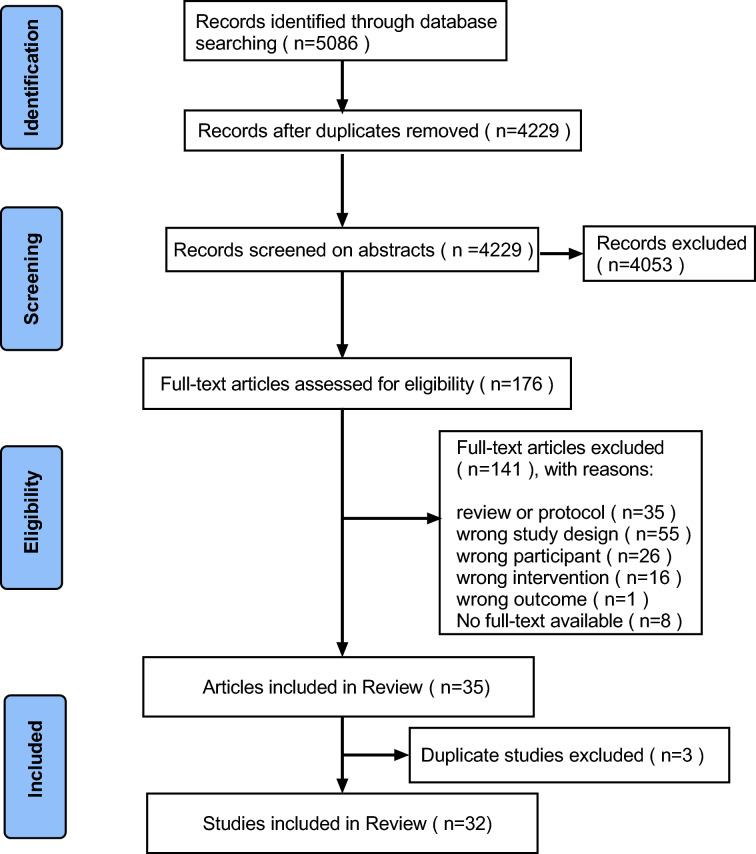
Flow chart of the literature search and screening process

Initially the database was searched for a total of 5086 studies, 4229 studies remained after eliminating duplicates, 4053 studies were excluded by reading the titles and abstracts. Subsequently, the full text of the remaining 176 studies was screened, excluding reviews or protocol (n = 35), wrong study design (n = 55), wrong participant (n = 26), wrong intervention (n = 16), wrong outcome (n = 1), No full-text available (n = 8). Finally, 35 articles were included in the review. 6 articles using the same intervention but reporting different outcomes were combined into 3 studies. The final number of included studies was 32. The specific flow chart is shown in Fig. 1.

### Descriptive Characteristics

The main characteristics of the included studies are summarized in Table 1. Thirty-two studies [22, 36–69] met the inclusion criteria. Publication years were from 1995 to 2023.

**Table 1:** Characteristics of included studies

	Study (Author)	Participants (age (years); diagnostic methods; Co-morbidity)	Sample size (Total number; intervention group/control group)	Intervention (Classification, Specifics)		Treatment duration	Outcome assessment				Results
				Experimental	Control		Standardised movement skills tests	Body function	Activity and participation	Psychosocial	
1	Cheng et al. [36]	6–9; DSM-V, MABC^a^; With ADHD, dyslexia or ASD	88;44/44	NMT(P)	Non-intervention	40 min/session 12 weeks 6 M Follow-up		Adaptive balance; Leg muscle activation			Failed to improve adaptive balance performance and leg muscle activation times
2	Coetzee and Pienaar [37]	7–8; DSM-V, MABC^a^	32;16/16	Visual therapy(P)	Non-intervention	40 min/session 1 session/week 18 weeks	MABC	Ocular motor control functions			Improve the ocular motor control functions (visual pursuit, fixation, ocular alignment and convergence–divergence)
3	EbrahimiSani et al. [38]	7–10; DSM-V, BOTMP-2^b^	40;20/20	VR (T)	Non-intervention	30 min/session 2 session/week 8 weeks 2 M Follow-up		Predictive motor control			Improved significantly on measures of MI, motor planning, and rapid and online control scores
4a	Fong et al. [39]	6–9; DSM-IV, MABC-2^a^; With ADHD, Dyslexia, AS, ASD	44;21/23	Taekwondo (T)	Non-intervention	60 min/session 1 session/week 12 weeks		Single-leg standing balance; Reactive balance control; Muscle strength			Improvements in isokinetic knee muscle strength at 180/s and static single-leg standing balance control, but do not benefit from improved reactive balance control
4b	Fong et al. [41]	6–9; DSM-IV, MABC-2^a^; With ADHD, Dyslexia, AS, ASD	44;21/23	Taekwondo (T)	Non-intervention	1 h/session 1 session/week 12 weeks		Sensory Organization Test; Single leg standing balance			Three months of daily TKD training can improve sensory organization and standing balance for children with DCD
5a	Fong et al. [40]	6–10; DSM-IV, MABC^c^/BOTMP; With ADHD, dyslexia or Suspected ASD	88;47/41	Task-specific FMT(T)	Non-intervention	1.5 h/session 2 session/week 12 weeks 3 M Follow-up	MABC	Sensory Organization Test			Improve the somatosensory function and somewhat improve the balance performance of children with DCD
5b	Fong et al. [43]	6–10; DSM-IV, MABC^c^/BOTMP; With ADHD, dyslexia or Suspected ASD	83;42/41	Task-specific FMT(T)	Non-intervention	1.5 h/session 2 session/week 12 weeks 3 M Follow-up		Standing balance; Muscle functions			MPT participants showed significant improvements in the balance strategies they adopted in the sensory challenge environment, as well as improvements in the knee extensor peak force and time to peak force in the knee flexors
6	Fong et al. [42]	9–12; DSM-V, MABC-2^b^; With ADHD, dyslexia or Suspected ASD	61;30/31	Tai chi(T)	Non-intervention	1.5 h/session 2 session/week 12 weeks	MABC-2	Limits of stability; Leg muscular performance	Fall history		Improvements in the peak forces of the knee extensors and flexors were demonstrated in the TC group
7	Gill et al. [44]	8–12; DSM-V, MABC-2^b^; With ADHD	78;39/39	CO-OP(T)	Wait-list	1 h/session 1 session/week 10 weeks	BOT-2		COPM; PQRS		Children with DCD showed improved motor outcomes following CO-OP
8	Green et al. [45]	5–11; DSM-V, MABC^a^	39;11/28	CO-OP(T)	Non-intervention	1 h/week 20 weeks	MABC				Children with severe DCD were likely to have continuing difficulties despite progress following intervention
9	Hashemi et al. [46]	7–11; DSM-V, MABC-2^b^	50;25/25	Wii Fit(T)	Usual school program	30 min/session 3 session/week 8 weeks		Visual-Perceptual Skills; Executive function			Wii Fit training improves visual perception and executive function in children with DCD
10	Hillier et al. [47]	5–8; DSM-IV; MABC^a^	12;6/6	Aquatic therapy(P, T)	Wait-list	30 min/session 1 session/week 6–8 weeks	MABC		Participation	PSPCSA	Aquatic physiotherapy may have benefits for motor skills
11	Izadi-Najafabadi et al. [48]	8–12; DSM-V, MABC-2^b^	37;17/20	CO-OP(T)	Wait-list	1 h/session 1 session/week 10 weeks 3 M Follow-up	BOT-2		COPM; PQRS		Intervention was effective for children with DCD in achieving and maintaining functional motor goals, as well as transfering of learning to other motor skills
12	Ju et al. [49]	5–10; DSM-V, MABC-2^b^	24;12/12	Video games(T)	Non-intervention	45 min/session 3 session/week 4 weeks		Static balance			iBalance training program is e®ective for a short time treatment e®ect for children with DCD
13	Kordi et al. [50]	7–9; DSM-IV, MABC^a^	30;15/15	Strength training(P)	Non-intervention	1 h/session 2 session/week 12 weeks		Isometric muscle strength; Static/Dynamic balance			The strength training leads to static balance improve in DCD children
14	Lichtsteiner et al. [51]	6–8; ICD-10, MABC-2^b^	42;22/20	PMI(P)	Wait-list	20 min/session 1 session/month 5 months			Fine Motor Performance; Handwriting		PMT has an effect on fine motor skills and some aspects of handwriting self-concept, but not handwriting fluency and consistency
15	Maharaj and Lallie [22]	6–12; MABC^a^	60;30/30	Gross motor programme(P, T)	Non-intervention	30 min/session 1 session/week 8 weeks	MABC		DCDQ		Improved motor function and activities of daily living in children with DCD
16	Marshall et al. [52]	7–11; MABC-2^c^, DCDQ	20;10/10	AO + MI(T)	Controlled intervention	21 session		Bimanual coordination performance			AO + MI group exhibited quicker completion times, more target-focused eye-movement behaviour and smoother movement kinematics compared to the control group
17	Navarro-Patón et al. [53]	4–6; MABC-2^c^	28;12/16	Physical education(T)	Non-intervention	40 min/session 1 session/week 6 weeks	MABC-2				Improvements in manual dexterity, aiming and catching and balance
18	Norouzi Seyed Hosseini et al. [54]	8–9; MABC^?^	40;20/20	QET(T)	Controlled intervention	40 min/session 1 session/week 4 weeks		Bimanual coordination performance			Bimanual coordination accuracy significantly increased
19	Peens et al. [55]	7–9; MABC^a^	28;11/17	PMI(P, T)	Non-intervention	motor-based intervention session: 30 min/2 session/weeks; 8 weeks psychological intervention session: 45 min/1 session/weeks; 8 weeks	MABC			TSCS-CF; CAS	Motor proficiency and self-concept of children with DCD benefit from intervention, but both should be addressed for optimal benefits
20a	Pless et al. [56]	5–6; DSM-IV, MABC^a^	37;17/20	Group motor skill intervention(T)	Non-intervention	20 min/session 1 session/week 10 weeks	MABC		MABC-C		Children with definite motor difficulties do not benefit from this type of intervention
20b	Pless et al. [57]	5–6; DSM-IV, MABC^a^	37;17/20	Group motor skill intervention(T)	Non-intervention	1 session/week 10 weeks	MABC			SPC	The intervention increased individual awareness of motor competence
21	Polatajko et al. [58]	7–13; BOTMP	50;26/24	Process-oriented treatment(P)	Non-intervention	12 session 5 weeks 6W Follow-up		Proprioceptive function; Visual-motor integration			The data suggest that an appropriate treatment strategy might be one that involves direct, repetitive training of a specific skill
22	Scott et al. [59]	7–12; DSM-V, MABC-2^c^	28;14/14	AO + MI(T)	Controlled intervention	40 min/session 1 session/week 4 weeks 2W Follow-up			ADL Performance times; ADL Technique ratings		AO-MI interventions can aid the learning of complex ADLs in children with DCD, and may be particularly effective for facilitating the learning of motor skills that do not currently exist within these children's motor repertoire
23	Sit et al. [60]	6–10; DSM, MABC-2^b^	69;35/34	FMT(T)	Non-intervention	40 min/session 1 session/week 8 weeks 3 M Follow-up	TGMD-2		Physical activity levels;	PSDQ	A school-based FMS training program has the potential to promote physical and psychological health in children with DCD in the long run
24	Straker et al. [61]	9–12; DSM-V, MABC^b^	21;10/11	Active electronic games(T)	Controlled intervention	20 min/session 4–5 session/week 16 weeks	MABC-2	Balance; hand–eye coordination	DCDQ		A 16 week home based AVG intervention did not enhance motor skills in children with DCD
25	Thornton et al. [62]	8–10; DSM-IV, MABC-2^?^	20;10/10	CO-OP(T)	Non-intervention	1 h/session 1 session/week 10 weeks	MABC-2		Handwriting Speed Test; Goal Attainment Scale; COPM		CO-OP is an approach which uses cognitive-based strategies to improve performance of specific tasks based on child chosen goals. The intervention program had a positive effect on self-perceived levels of performance which may lead to changes in quality of life. Parents felt the intervention enhanced socialisation, peer modelling and encouragement and felt that this increased confidence and independence
26	Tsai, 2009 [63]	9–10; DSM-IV, MABC^c^	28;14/14	Table-tennis training( T)	Non-intervention	50 min/session 3 session/week 10 weeks	MABC	Inhibitory control			Table-tennis training resulted in significant improvement of cognitive and motor functions for the children with DCD
27	Tsai et al. [64]	11–12; DSM-IV; MABC-2^a^	40;20/20	Chronic aerobic exercise(T)	Non-intervention	50 min/session 3 session/week 16 weeks	MABC-2	Cardiorespiratory fitness; Visuospatial working memory			Increased cardiorespiratory fitness could effectively improve the performance of the VSWM task in children with DCD, by enabling the allocation of greater working memory resources related to encoding and retrieval
28	Tsai et al. [65]	9–10; DSM-IV, MABC^c^	30;16/14	Soccer training(P, T)	Non-intervention	50 min/session 5 session/week 10 weeks	MABC	Attention network			Soccer training resulted in significant improvements in ERP and task performance indices for the children with DCD
29	Tseng et al. [66]	9–10; DSM-V, MABC-2^b^	20;10/10	Table-tennis training(T)	Non-intervention	40 min/session 3 session/week 12 weeks	MABC-2	Haptic Function			41.5% improvement in haptic sensitivity in children exposed to table tennis training
30	Wilson et al. [67]	7–12; DSM-V, MABC^d^	22;11/11	Motor imagery training(T)	Wait-list	60 min/session 1 session/week 5 weeks	MABC				Improved motor performance significantly
31	Wood et al. [68]	7–11; MABC-2^c^	21;11/10	QET + TT(T)	TT	4 weeks 2 weeks Follow-up			Catching performance		Children improved their gaze control and catching coordination following QET
32	Yu et al. [69]	7–10; DSM-IV, MABC-2^a^	38;22/16	FMT(T)	Non-intervention	35 min/session 2 session/week 6 weeks	TGMD-2		Physical activity Levels		FMS training is effective in improving fundamental movement skills and self-perceived physical competence and reducing sleep disturbances for children with DCD

*AO+MI* Action observation and motor imagery, *ADHD* Attention deficit hyperactivity disorder, *AS* Asperger’s Syndrome, *ASD* Autism spectrum disorder, *BOTMP* Bruininks-Oseretsky Test of Motor Proficiency, *CAS* Child Anxiety Scale, *CO-OP* Cognitive Orientation to (Daily) Occupational Performance, *COPM* Canadian Occupational Performance Measure, *DCDQ* Developmental Coordination Disorder Questionnaire, *FMT* functional-movement training, *MABC* Movement Assessment Battery for Children Test, *MABC-C* Movement Assessment Battery for Children–Checklist, *NMT* Neuromuscular training, *P* process-oriented, *PMI* Psycho-motor intervention, *PSDQ* Physical Self-Descriptive Questionnaire, *PSPCSA* Pictorial Scale for Perceived Competence and Social Acceptance for Young Children, *PQRS* Performance Quality Rating Scale, *T+P* Combined task- and process-oriented, *QET* Quiet eye training, *SPC* Self-perceived competence, *T* task-oriented, *TGMD-2* Test of Gross Motor Development-2, *TSCS-CF* Tennessee Self-Concept Scale (Child Form) , *VR* Visual therapy, *?* not reported or unclear

^a^≤ 15th percentile

^b^≤ 16th percentile

^c^≤ 5th percentile

^d^< 10th percentile

#### Participants

A total of 1265 participants were included across the studies, with 624 assigned to the intervention group and 641 to the control group. Participant ages ranged from 4 to 13 years. Twenty-five studies explicitly reported the use of diagnostic criteria (DSM-IV/V or ICD-10) for diagnosing DCD, seven studies utilized standardized motor tests (MABC 1/2, BOTMP) to assess Criterion A and confirm motor impairments in children, thus identifying them as p-DCD.

#### Interventions

The interventions in the treatment group were all motor-based intervention. categorized as process-oriented interventions (n = 6, 18.8%), task-oriented interventions (n = 21, 65.6%), combined task- and process-oriented interventions (n = 5, 15.6%), and the duration of the intervention ranged from 4 weeks to 5 months. In the control group, twenty-one studies received no intervention, five were on a wait-list control, five were on an active controlled intervention, and one were assigned to passive controls.

#### Outcomes

The outcome indicators consisted of four broad categories. They were: (1) Standardised movement skills tests (n = 17); (2) Body functions: balance function (n = 7); cognitive function (n = 5); muscle function (n = 3); coordination function (n = 3); visual Function (n = 3); sensory function (n = 2); sensory organization function (n = 2); (3) activities and participation (n = 9); (4) Psychosocial factors (n = 3).

### Risk of Bias

**Fig. 2:**
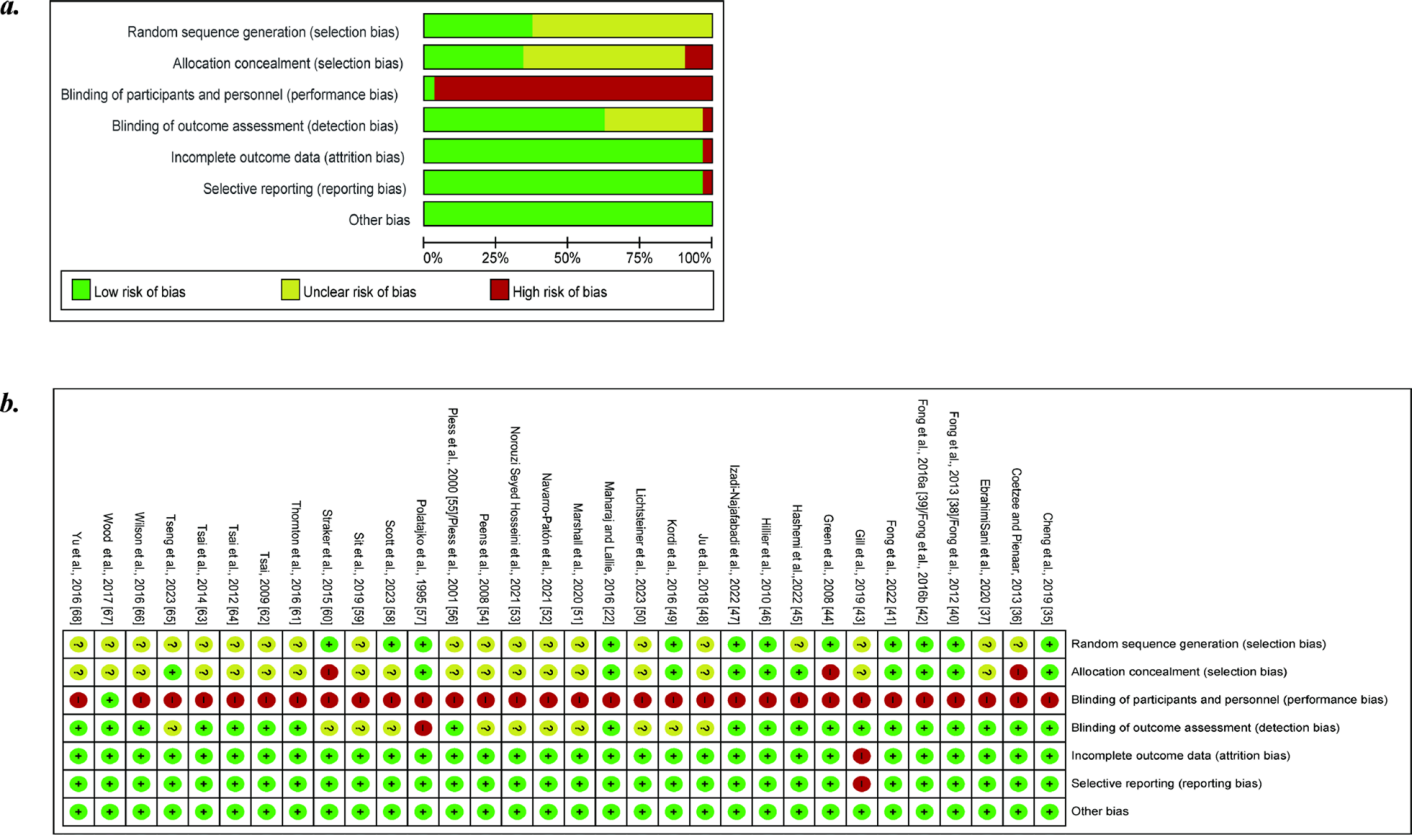
Summary of the risk of bias assessment. **a** Risk of bias graph: review authors' judgements about each risk of bias item presented as percentages across all included studies; **b** Risk of bias summary: review authors' judgements about each risk of bias item for each included study

Of the thirty-two studies, seven (22%) had a low risk of bias on more than six criteria, five (16%) had a low risk of bias on five criteria, twelve (37%) had a low risk of bias on four criteria, seven (22%) had a low risk of bias on three criteria, and one (3%) had a low risk of bias on two criteria. Regarding the risk of randomized sequence generation, all studies were randomly assigned, but only twelve studies reported a reasonable method of randomization, which was judged to be "Low risk", while the remaining twenty studies did not explicitly mention randomization and were judged to be "Unclear risk". Eleven trials that explicitly described the method of allocation concealment had a low risk of bias for allocation concealment, three studies that did not appropriately conceal allocations had a high risk of bias, and eighteen trials had an unclear risk of bias. One study mentioned that children and their parents were blinded to their intervention allocation, judged to be at low risk. All remaining studies did not blind participants and were at high risk of bias. Twenty trials used an independent assessor with a low risk of detection bias, one trials used an intervener for the assessment with a high risk of detection bias, and the remaining eleven studies did not explicitly mention an assessor. One studies were deemed to have a high risk of bias due to incomplete outcome data and selective reporting, while the remaining studies were considered to have a low risk. No other sources of bias were detected in any of the trials, resulting in an overall judgment of low risk of bias in this domain for all studies. A summary of our assessment is shown in Fig. 2.

### Meta-analysis

The present meta-analysis incorporated the standardized movement skills test as the primary outcome measure, alongside secondary assessments including body functions, activity and participation levels, and psychosocial factors. Table 2 provides a summary of the findings for each outcome.

**Table 2:** Summary of each outcome's results

Information	N	P	g	*p*-value	Q	*I*-squared
Standardised Movement Skills Tests	17	621	1.00	< 0.001**	141.89	88.82
Task-oriented	12	459	0.57	0.007**	49.91	77.96
Combined task- and process-oriented	4	130	1.53	0.038*	35.28	91.50
Gross Motor Skills	6	257	0.95	0.04*	53.46	90.65
Fine Motor Skills	4	109	0.28	0.61	21.70	86.17
Hand–eye coordination skills	5	169	1.14	0.03*	35.82	88.83
Body functions						
Balance function	7	334	0.57	0.005**	18.21	67.04
Process-oriented	2	118	0.15	0.407	0.07	0.00
Task-oriented	5	216	0.76	0.003**	11.66	65.69
Cognitive function	5	188	1.53	0.001**	27.56	85.49
Muscle function	3	174	0.91	0.017*	10.26	80.51
Coordination function	3	81	0.47	0.032*	1.77	0.00
Visual Function	3	132	0.61	0.009**	3.54	43.45
Sensory function	2	70	0.85	0.001**	1.07	6.86
Sensory organization function	2	132	0.61	0.001**	0.89	0.00
Activity	8	329	0.71	0.004**	29.75	76.47
Task-oriented	6	215	0.71	0.014*	20.42	75.52
Psychosocial factors	3	77	0.71	0.079	5.31	62.36

**p < 0.01, *p < 0.05, N = Number of Studies, P = Number of participants; g = Hedges' g; Q = Cochran’s Q

#### Primary Outcomes

##### Standardised Movement Skills Tests

**Fig. 3:**
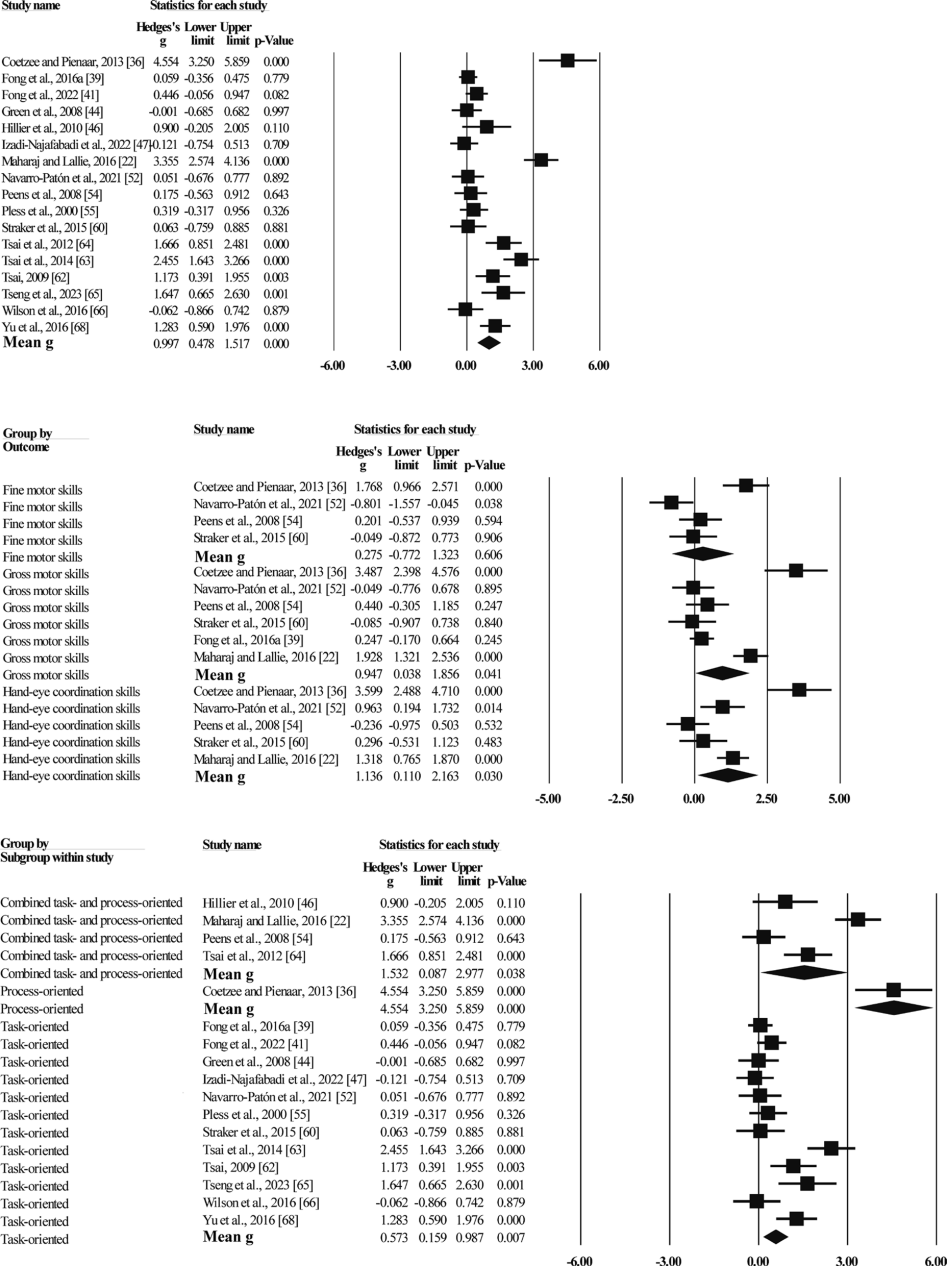
Forest plot of meta-analysis of standardised movement skills tests

Nineteen studies using standardized motor skill tests (e.g., MABC, MABC-2, BOT-2, TGMD-2) as an indicator of outcome with a total of 621 participants (see Fig. 3). the effect sizes ranged from g = − 0.12 to g = 4.55 and the pooled effect size was positive and significant (g = 1.00, N = 17, 95%CI [0.48,1.52], p < 0.001), MBI significantly improved children's standardized motor skill test scores. However, there was a high degree of heterogeneity between these studies (Q = 141.89, I^2^ = 88.82). Subgroup analyses with skill outcomes as a group showed that MBI significantly improved DCD children's gross motor skills (g = 0.95, N = 6, 95%CI [0.04,1,86], p = 0.041) and hand–eye coordination skills (g = 1.14, N = 5, 95%CI [0.11,2.16], p = 0.03), but the effect on fine motor skills (g = 0.28, N = 4, 95%CI [− 0.77,1.32], p = 0.606) was not significant. Heterogeneity ranged from 86 to 91%. Subgroup analyses by intervention type showed that task-oriented (g = 0.57, N = 12, 95%CI [0.16,0.99], p = 0.007) and combined task- and process-oriented (g = 1.53, N = 4, 95%CI [0.09,2.98], p = 0.038) training significantly improved standardized motor skill test scores in children with DCD. Heterogeneity was 78% and 92%, respectively.

#### Secondary Outcomes

##### Body Functions

**Fig. 4:**
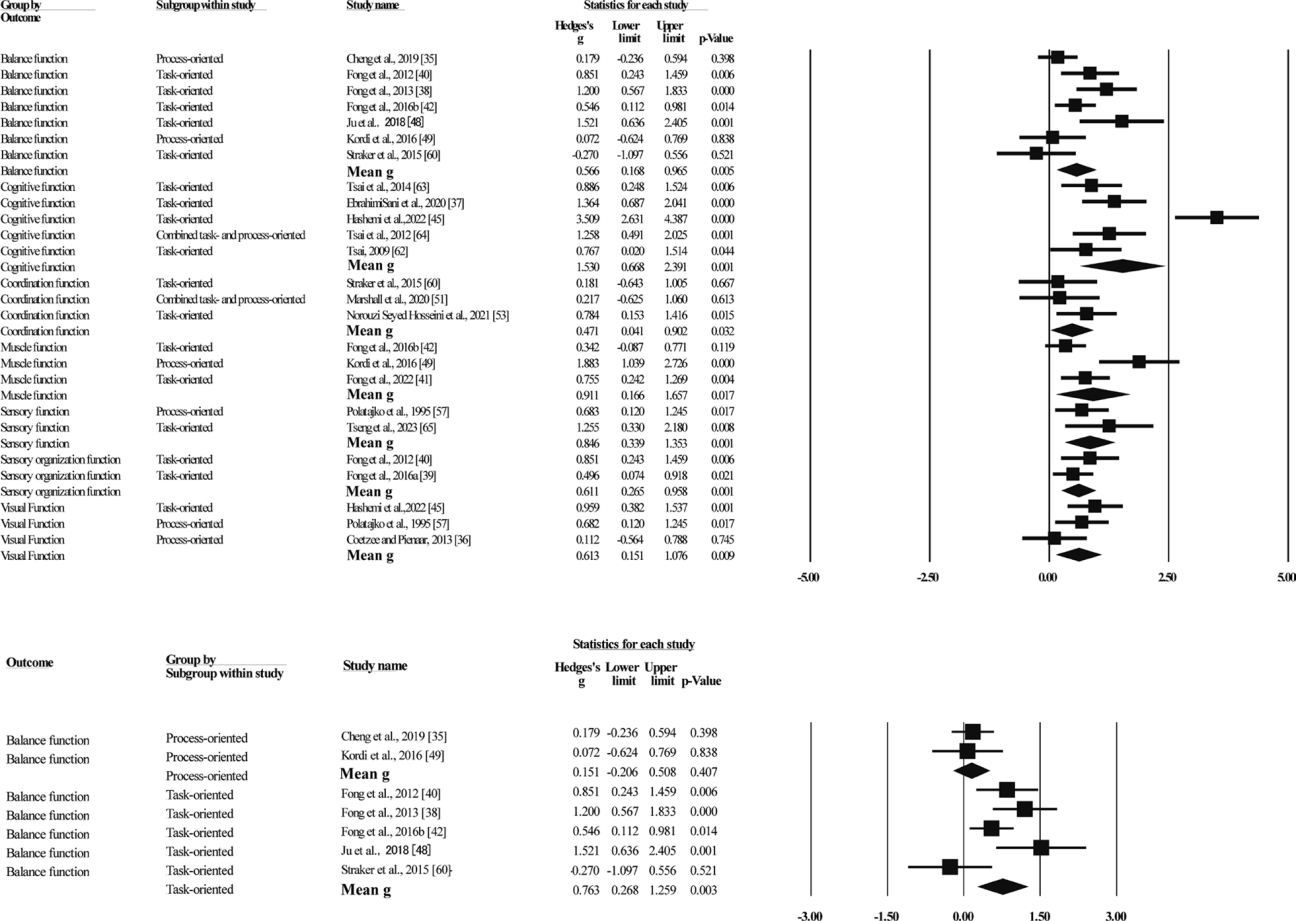
Forest plot of meta-analysis of body functions

Improvements in body functions showed that MBI significantly improved balance function (g = 0.57, N = 7, 95%CI [0.17,0.97], p = 0.005), cognitive function (g = 1.53, N = 5, 95%CI [0.67,2.39], p = 0.001), muscle function (g = 0.91, N = 3, 95%CI [0.17,1.66], p = 0.017), coordination function (g = 0.47, N = 3, 95%CI [0.04,0.90], p = 0.032), visual function (g = 0.61, N = 3, 95%CI [0.15,1.08], p = 0.009), sensory function (g = 0.85, N = 2, 95%CI [0.34,1.35], p = 0.001), and sensory organization function (g = 0.61, N = 2, 95%CI [0.27,0.96], p = 0.001) in children with DCD (see Fig. 4). Heterogeneity ranged from 0 to 85%. Due to the limitation of the number of included studies, only subgroup analyses were conducted for balance function and cognitive function.

Balance function subgroup analyses showed that task-oriented training (g = 0.76, N = 5, 95%CI [0.27,1.26], p = 0.003) significantly improved balance function in children with DCD, but process-oriented training (g = 0.15, N = 2, 95%CI [− 0.21,0.51], p = 0.407) showed no significant improvement, with heterogeneity of 66% and 0%, respectively.

##### Activities and Participation

**Fig. 5:**
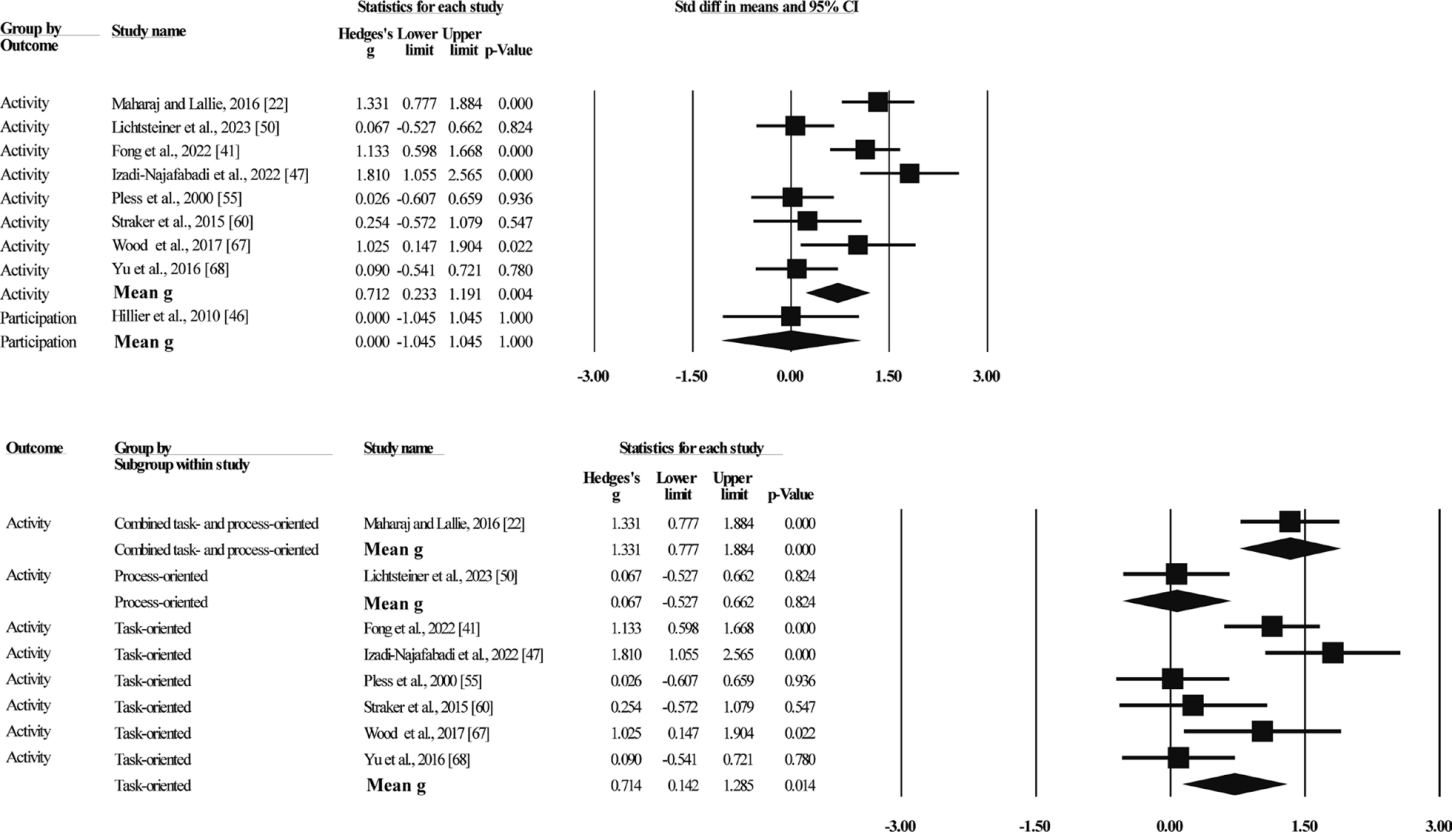
Forest plot of meta-analysis of activities and participation

Nine studies reported the effects of MBIs on activity and participation levels of children with DCD in terms of walking performance, history of falls, writing, and social participation through subjective-report questionnaires, objective activity tests, and direct behavioral observations of the children (see Table 1 for details), with a total of 401 participants (see Fig. 5). Of these, eight studies [22, 42, 48, 51, 56, 61, 68, 69] (389 participants) reported results at the activity level, and one study [47] (12 participants) reported results at the participation level. The results of the activity level meta-analysis indicated that the effect sizes ranged from g = 0.03 to g = 1.81, and that MBI significantly improved activity levels in children with DCD (g = 0.71, N = 8, 95%CI [0.23,1.19], p = 0.004). Heterogeneity (Q = 29.75, I^2^ = 76). Subgroup analyses revealed that task-oriented (g = 0.71, N = 6, 95%CI [0.14,1.29], p = 0.014) training significantly improved DCD children's activities performance, with 76% heterogeneity. One study each reported improvements in activity levels with process-oriented (g = 0.07, p = 0.824) as well as combined task- and process-oriented (g = 1.33, p < 0.001) training. One study reported results for participation levels (g = 0.00, p = 1.00).

##### Psychosocial Factors

**Fig. 6:**
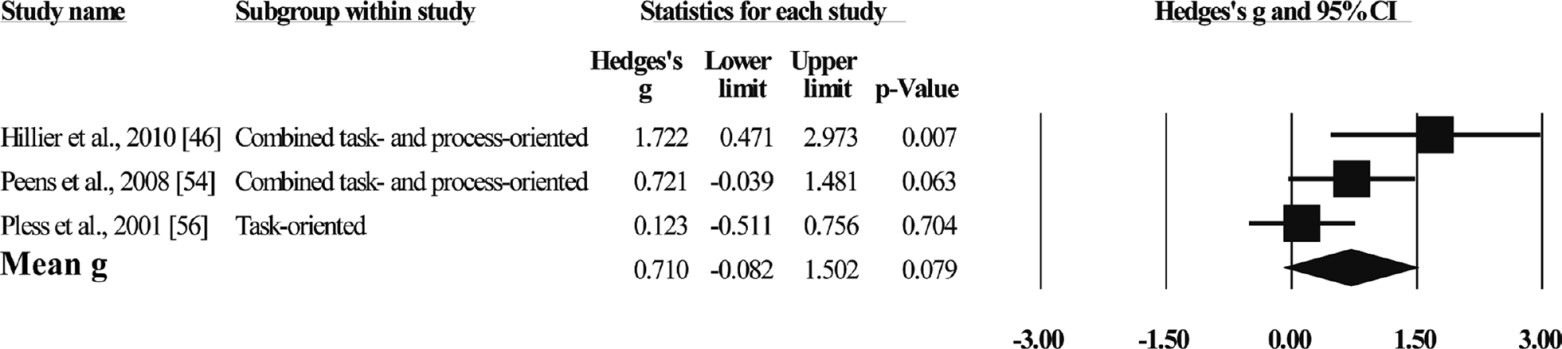
Forest plot of meta-analysis of psychosocial factors

Four studies reported improvements in Psychosocial factors with a total of 77 participants (see Fig. 6). The effect sizes ranged from g = 0.12 to g = 1.72, with no statistically significant difference in the combined effect size (g = 0.71, N = 3, 95%CI[-0.08,1.50], p = 0.079). The results suggest that MBI failed to improve the psychosocial status of children with DCD. Heterogeneity (Q = 5.31, I^2^ = 62).

### Certainty of Evidence (GRADE)

Using the GRADE system recommended methodology, we assessed the quality of evidence for the study outcomes. The evidence for standardized motor skills tests and balance function was rated as "moderate" due to heterogeneity between studies. Cognitive function and muscle function were rated as "low" because of both between-study heterogeneity and the small sample sizes in the included studies. Coordination function and sensory integration function were rated as "moderate" due to the small sample sizes of the included studies. Visual function and sensory function were rated as "low" because of potential risk of bias in the studies and the small sample sizes. Activity level was rated as “moderate” due to the risk of bias between studies. Social–psychological factors were rated as "low" due to the 95% confidence interval crossing the threshold and the small sample sizes in the studies. Table 3 provides a summary of the certainty of available evidence.

**Table 3:** Summary of the certainty of available evidence

Outcome	Quality assessment							No of patients		Effect	Quality
	No of studies	Design	Risk of bias	Inconsistency	Indirectness	Imprecision	Other considerations	Experimental	Control	Absolute	
Standardised Movement Skills Tests	17	Randomised trials	No serious risk of bias	Serious^1^	No serious indirectness	No serious imprecision	None	318	303	Hedges' g 1.00 (0.48 to 1.52)	⊕⊕⊕⊝ MODERATE
Balance function	7	Randomised trials	No serious risk of bias	Serious^1^	No serious indirectness	No serious imprecision	None	165	169	Hedges' g 0.57 (0.17 to 0.97)	⊕⊕⊕⊝ MODERATE
Cognitive function	5	Randomised trials	No serious risk of bias	Serious^1^	No serious indirectness	Serious^2^	None	95	93	Hedges' g 1.53 (0.67 to 2.39)	⊕⊕⊝⊝ LOW
Muscle function	3	Randomised trials	No serious risk of bias	Serious^1^	No serious indirectness	Serious^2^	None	87	87	Hedges' g 0.91 (0.17 to 1.66)	⊕⊕⊝⊝ LOW
Coordination function	3	Randomised trials	No serious risk of bias	No serious inconsistency	No serious indirectness	Serious^2^	None	40	41	Hedges' g 0.47 (0.04 to 0.9)	⊕⊕⊕⊝ MODERATE
Visual function	3	Randomised trials	Serious^3^	No serious inconsistency	No serious indirectness	Serious^2^	None	67	65	Hedges' g 0.61 (0.15 to 1.08)	⊕⊕⊝⊝ LOW
Sensory function	2	Randomised trials	Serious^4^	No serious inconsistency	No serious indirectness	Serious^2^	None	36	34	Hedges' g 0.85 (0.34 to 1.35)	⊕⊕⊝⊝ LOW
Sensory organization function	2	Randomised trials	No serious risk of bias	No serious inconsistency	No serious indirectness	Serious^2^	None	68	64	Hedges' g 0.61 (0.27 to 0.96)	⊕⊕⊕⊝ MODERATE
Activity	8	Randomised trials	No serious risk of bias	Serious^1^	No serious indirectness	No serious imprecision	None	159	158	Hedges' g 0.71 (0.23 to 1.19)	⊕⊕⊕⊝ MODERATE
Psychosocial factors	3	Randomised trials	No serious risk of bias	No serious inconsistency	No serious indirectness	Very serious^5^	None	34	43	Hedges' g 0.71 (− 0.08 to 1.50)	⊕⊕⊝⊝ LOW

^1^Heterogeneity across studies

^2^The sample size of included studies was low

^3^1 studies did not appropriately conceal allocation, 1 study outcome assessment was not blinded

^4^1 study outcome assessment was not blinded

^5^95% CI crosses the threshold, and the sample size of included studies was low

## Discussion

### Summary of Results

We conducted the systematic meta-analysis of available data, encompassing a total of 32 RCTs, to assess the effectiveness of various types of MBIs in children with DCD, including those with probable DCD. The study results indicated that MBIs significantly improves overall motor skills, body functions, and activity performance in children with DCD. However, no improvement in children's level of participation was observed, nor were significant improvements observed in children's psychosocial factors.

### Effect of MBI on Overall Motor Skills in Children with DCD

In this study, we utilized a standardized motor skill test to assess children's overall motor skills. According to the results of the meta-analysis, MBI led to a significant improvement in overall motor skills (g = 1.00, p < 0.001), indicating the effectiveness of this intervention in enhancing the motor skills of children with DCD. However, there was considerable overall heterogeneity (I^2^ = 88.82), which may be attributed to variations in the specific interventions and their durations. Additionally, the differing selection criteria used across studies represent a significant source of this heterogeneity. Because of the presence of heterogeneity, the certainty of the evidence for the standardized motor skill test GRADE is moderate. Further analysis indicated that MBI were effective in enhancing gross motor skills (g = 0.95, p = 0.04) and hand–eye coordination skills (g = 1.14, p = 0.03) in children with DCD. However, there was no significant improvement observed in children's fine motor skills (g = 0.28, p = 0.61). This outcome is not surprising, as very few interventions in the studies examined to date focused on improving fine motor skills [62, 70]. In the four studies reporting fine motor skills, two studies [53, 55] included interventions specifically targeting fine motor activities aimed at improving children's hand dexterity, the remaining two studies [37, 61] did not focus on fine motor skill training, this may have contributed to a lower overall effect size and potentially resulted in the loss of statistical significance in the final pooled analysis. Additionally, achieving desirable rehabilitation outcomes for fine motor skills typically necessitates longer intervention periods compared to other motor skills [3, 71]. The intervention sessions in the studies included thus far are relatively brief (Minimum 6 weeks, maximum 18 weeks) [37, 55], potentially insufficient to yield a positive effect on fine motor skills. Therefore, to enhance fine motor skills in children, it is essential to incorporate more activities specifically targeting hand-based tasks in the chosen interventions. Additionally, it is advisable to extend the duration of the intervention as much as possible to achieve more significant improvements.

The findings from previous studies have suggested that task-oriented motor training is effective in enhancing the overall motor skills of children with DCD [9, 19, 72, 73]. This study further corroborates this notion, as we observed that the effectiveness of task-oriented MBI on the overall motor skills of children with DCD was significant, demonstrating a moderate effect size (g = 0.57, p = 0.005). From the perspective of brain plasticity, individuals learn and acquire skills most effectively when they perceive the training as relevant and beneficial to their lives [74]. Task-oriented training directs attention towards mastering specific tasks [9], taking into full consideration children's motivation to learn. In contrast, process-oriented approaches concentrate on rectifying underlying deficits and improving motor performance by addressing functional aspects [24, 75], often without effectively engaging children's motivation. Additionally, task-oriented training prompts children to contemplate the nature of the challenges encountered during tasks and to devise solutions, thereby enhancing task-specific performance at a cognitive level and proving more effective in honing children's skills. With only 1 study [37] reporting process-oriented training and presenting a high effect size a limited number of studies, the intervention effect of process-oriented training on overall motor skills is still inconclusive and needs to be further validated by more studies in the future. Meanwhile, the combined task- and process-oriented approach (four studies) [22, 47, 55, 65] also significantly enhanced children's motor skills, with a larger effect size (g = 1.53, p = 0.038) than task-oriented training alone. This finding aligns with previous studies [19], suggesting that positively focusing on task performance and further enhancing the improvement of children's functional status can contribute to the overall improvement of motor skills in children with DCD to a greater extent, and this multilevel approach warrants further investigation and dissemination in the future.

### Effect of MBI on Body Functions in Children with DCD

The impact of MBI on body functions encompasses several facets. Our findings show that MBI significantly enhances balance function, cognitive function, muscle function in children with DCD. In further subgroup analyses, task-oriented exercise training significantly improved balance function in children with DCD (g = 0.76, p = 0.003), while process-oriented training had no significant effect (g = 0.15, p = 0.407). The intrinsic mechanisms of balance function are complex [76, 77], and in addition to basic biomechanical mechanisms, maintaining balance is closely intertwined with higher cognitive functions, such as executive function [78], therefore, the effect of remedying underlying process deficits in impaired function may not be significant by focusing training only on the impaired function itself, as in the case of single process-oriented training such as neuromuscular/strength training, whereas Task-oriented training employs child-centered cognitive strategies that emphasize the interaction between cognitive and motor abilities [79, 80], as in the case of trampoline training, where the child's interest can be sufficiently mobilised to seek out the balance difficulties encountered during a trampoline task and find solutions to them, which may have a more significant effect on improving balance function. Balance responses are closely related to posture and task demands, which may explain why different interventions lead to varying effects. Our study also validated the effect of MBI on improving cognitive functioning in children with DCD (g = 1.53, p = 0.001), and the training involved all included task-oriented strategies (four [38, 46, 63, 64] purely task-oriented, and one [65] combined task- and process-oriented), whereas no study has yet reported on the effect of process-oriented training on improving cognitive function. The improved cognitive function primarily encompasses executive functioning, attentional networks, and predictive motor control, with measurement tools such as the visuospatial working memory paradigm for attentional tasks [64, 65], the cognitive assessment system to measure executive function [46], and the motor imagery tests, action planning tests, and rapid online control tests were used to assess predictive motor control [38]. The intervention type involves activities that necessitate quick reactions and responses from children, such as soccer, ping-pong, and other ball games [63, 65], as well as active video games [38, 46].

Furthermore, MBI significantly enhanced muscle function (g = 0.91, p = 0.017), coordination function (g = 0.47, p = 0.032), visual function (g = 0.61, p = 0.01), sensory function (g = 0.85, p = 0.001), and sensory organization function (g = 0.61, p = 0.001) in children with DCD. However, due to limitations in the literature, we were unable to conduct subgroup analyses based on interventions as categories for these aspects. Nevertheless, we found that three studies reported improvements in muscle function, Kordi's study used process-oriented direct strength training [50] and two studies [40, 42] used task-specific functional training. Three studies reported coordination functions (including bimanual coordination function and hand–eye coordination function) [52, 54, 61], all involved task-oriented training. Conversely, no single process-oriented training study has reported an effect on coordination function, thus, its efficacy cannot be determined. Three studies [37, 46, 58] reported the effects of MBI on visual functions in children with DCD, encompassing ocular motor control function, visual perception function, and visual-motor integration function. Visual function plays a crucial role in the development of motor skills, particularly in visuospatial perception and visuomotor skills [81–83]. These skills are closely associated with the execution of bilaterally coordinated activities and various fine manipulations [84]. Two studies utilized virtual reality system to enhance visual functioning in children with DCD. By creating a sensory illusion through virtual reality, these interventions enabled children to perceive the virtual environment as reality and engage in rich motor activities in an immersive manner [85]. Consequently, this expanded the visual attention pool and enhanced visuospatial perception as well as visuomotor adaptation. In other reported studies [54, 86], quiet eye training appears to be an effective visual-perceptual training program for enhancing visual-motor control. Additionally, two studies [58, 66] highlighted improvements in sensory function: Polatajko's research demonstrated that process-oriented training was effective in enhancing kinesthetic acuity and accuracy of discrimination of upper limb movement and position (deep sensory function) in children with DCD. Tseng reported improvements in tactile perception (superficial sensory function) with table tennis training, and noted that the sensory enhancement coincided with changes in motor function in children with DCD. It is evident that MBI can exert a positive and beneficial effect on both superficial and deep sensations in children. However, it should be noted that conclusions drawn from a single study are only preliminary, and the actual effect requires further validation in future research. Regarding sensory organization function (all balance tasks), both studies utilized task-oriented training, effectively improving the somatosensory ratio [40], vestibular ratio, and vestibular ratio [41] in children with DCD. However, the GRADE evidence certainty for body function was moderate or low due to heterogeneity among studies or the sample size of included studies was too small, more studies are needed in the future to increase the sample size and implement more stringent inclusion criteria to enhance the level of evidence.

### Effects of MBI on Activity and Participation Levels in Children with DCD

We also reported improvements in MBI on activity performance in children with DCD (g = 0.71, p = 0.004). As MBI enhances children's motor skills, there is a corresponding improvement in their performance of activities. However, due to heterogeneity between studies, GRADE evidence certainty was moderate. Further studies revealed that task-oriented (g = 0.71, p = 0.014) MBIs significantly enhanced children's activity levels, demonstrating large effect sizes. However, the studies were moderately heterogeneous, probably because of the different tasks (such as running, jumping, catching, kicking and throwing for functional movement training tasks [69] selected in the specific intervention methods, which brought about different levels of intervention effects. Task-oriented training aims to encourage children's active participation in activities, focusing on interventions for daily activity skills [20]. It targets specific tasks within children's everyday situations and emphasizes the extension of learned daily functions and activities to various environments where children need to complete them. This approach aims to improve children's participation skills in a holistic manner. Furthermore, task-oriented training aids the child in gaining a deeper understanding of the task requirements, clarifying the process necessary for goal achievement, and identifying the steps needed to execute the movement. It intervenes in the dimension of cognitive strategies for the child's selected goal, thereby enhancing the performance of a specific task [80]. With the improvement of cognitive strategies, they can be applied to other areas of daily life, leading to positive transfers in similar tasks [79, 87]. One study reported the effects of process-oriented training (g = 0.07, p = 0.824) on children's activity levels, while another study examined the impact of combined task- and process-oriented training (g = 1.33, p < 0.001). Based on these findings, the current strategies for improving activity performance in children with DCD primarily focus on task-oriented training. In task-oriented interventions, the focus was on teaching children functional tasks. The interventions aimed to concentrate on executing specific motor tasks or 'occupations,' such as tying shoelaces, catching a ball, and handwriting, etc.. These tasks were designed with varying levels of difficulty and strategies to target specific activities that children struggled with, with the goal of improving relevant skills.

Only 1 study (12 participants) [47] reported results on the level of participation (g = 0.00, p = 1.00), which presented a negative result, but that study included fewer participants (12 participants) and a shorter intervention period (30 min/session; 1 session/week; 6–8 weeks), so the result was unstable. Due to the limitations of the amount of available literature, more research is needed in the future to explore the effects of MBI on the participation levels of children with DCD, and improving children's ability to participate in activities of daily living remains the ultimate goal of interventions for children with DCD. Furthermore, it is important to note that increasing children's participation in real-life situations necessitates considering the physical, social, and cultural environmental factors relevant to children's lives. This involves removing any barriers to participation [5] and actively obtaining support from children's parents and teachers. By doing so, children are provided with ample opportunities to practice motor skills and engage in daily activities [2].

### Effects of MBI on Psychosocial Factors in Children with DCD

Unfortunately, our study did not find significant improvements in psychosocial factors in children with DCD with MBI (g = 0.71, p = 0.079). However, the certainty of evidence for psychosocial GRADE was low due to the 95% CI crosses the threshold and the small sample size of included studies. It is also possible that the effects of the MBI on psychosocial factors may take more time and become apparent after a certain period of time after the end of the intervention. Whereas the measurements in the studies we included were taken directly after the end of the intervention, the effects may not yet be evident. In terms of psychomotor theory, as children's motor skills improve, their self-concept and self-efficacy increase, thereby fostering positive self-evaluation [21]. Furthermore, as children experience success and mastery in accomplishing motor tasks, they are likely to see a reduction in emotional barriers such as anxiety and depression [88, 89]. Increased motor competence can result in reduced resistance to interacting with peers, facilitating the development of social skills. It follows that while the current evidence is insufficient to clarify the efficacy of MBI in terms of psychosocial factors in children with DCD, this represents a highly promising direction for future research. Social cognitive theory suggests that higher self-efficacy is associated with improved activity and engagement performance [90]. The intervention model addressing children's social and psychological factors should be tailored to accommodate individual differences in motor abilities. The focus should be on promoting a sense of success and enjoyment during participation in training activities. Intervention strategies should involve activities that capture the children's interest, thereby increasing their motivation to engage in training. The environment should be one where children can feel fully relaxed and engaged, ensuring that they enjoy the training experience. Training tasks should be challenging but achievable, minimizing errors during practice to help children experience a sense of mastery and accomplishment [60].

## Strengths and Limitations

This review possesses several strengths. The included literature comprises studies with a RCT design, which investigated the effects of MBI on overall motor skills, body functions, activity and participation levels, and psychosocial factors in children with DCD. Additionally, it identified the specific effects of different categories of interventions. However, this systematic review has some limitations. Most of the meta-analyses exhibited moderate to high heterogeneity, which could be attributed to several factors. These may include variations in intervention methods (such as type of MBI, intervention duration, session duration, and frequency) or differences in the variables included in the quantitative analyses (i.e., clinical diversity). Additionally, six studies used only standardized motor tests to assess motor impairments in children, but did not report the diagnostic criteria (DSM-IV/V) employed, thus only categorizing these children as p-DCD. Therefore, stricter inclusion criteria are crucial, as including clinically diagnosed DCD children, as well as those with more severe DCD (< 5th percentile), may lead to different results. Some studies [36, 39–44] also reported co-morbidities (e.g., ADHD, ASD, Dyslexia, etc.) in children with DCD, which may be a possible source of partial heterogeneity. Secondly, the effects of MBI on psychosocial factors are derived from a relatively small number of studies and may be susceptible to publication bias. Finally, due to the paucity of research, we were unable to determine the effect of MBI on participation levels of children with DCD and the effects of process-oriented training on children's overall motor skills as well as activity levels.

## Recommendations for Practice and Future Research

The present study found that MBIs significantly improve overall motor skills, body function, and activity levels in children with DCD. However, no improvements were observed in the children's participation abilities. Further analysis revealed that task-oriented training had a significant positive effect on overall motor skills, body function, and activity levels. Due to the limited number of studies, the impact of process-oriented training on overall motor skills could not be conclusively determined. On the other hand, the combined task- and process-oriented strategies was found to significantly enhance overall motor skills in children with DCD. These findings further support the recommendations and statements outlined in the 2019 DCD International Guidelines [2]: If an intervention is to be provided then we recommend that activity‐oriented and participation-oriented approaches be used as a means to improve general, fundamental, and specific motor skills in individuals with DCD (Recommendation 22); Some interventions that aim to improve body functions and structures may be effective, but there is limited evidence whether body function-oriented interventions are effective in improving activity and participation in children with DCD (Statement 2). Additionally, our study recommends the use of a multi-level approach, which combines functionally oriented therapies with other activity-based therapies (e.g., strength training, perceptual training with specific functional tasks) to enhance the overall motor skills of children with DCD. In clinical practice, several crucial elements need to be considered: Firstly, individualised characteristics and their focus on everyday functional tasks are core elements of task-oriented training, tasks selected should be customized to match each child's characteristics and closely linked to their daily activity challenges, individually adapted to the specific task difficulties faced by the child, and with an emphasis on factors such as task difficulty and strategy selection. Secondly, the primary aim of task-oriented interventions is to enhance children's activity and participation levels. Therefore, interventions must be goal-oriented to spark children's interest and encourage their active involvement. Thirdly, active involvement of caregivers is crucial during training to ensure learning is transferred to the child's daily life. This involvement fosters positive transfer during similar tasks, enhancing the overall training impact. Lastly, in specific clinical settings, intervention focusing on environmental factors is essential. Throughout the treatment process, parents, teachers, and other significant individuals should provide necessary environmental support. Through collaborative efforts, a conducive external environment can be created for the child, promoting better motor skill acquisition and social participation. The current evidence did not reveal significant improvement in MBI on psychosocial factors in children with DCD. However, several studies [47, 55, 57] support the enhancement of self-efficacy and alleviation of various mood disorders in children through motor training. The limited number of studies contributes to many of the negative results. Therefore, we still recommend employing MBI to enhance the psychosocial well-being of children with DCD in clinical settings.

Our study has also highlighted numerous unanswered questions in this domain: For instance, whether MBI can improve children's participation levels further than it improves their activity levels, and whether process-oriented MBI can improve the overall motor skills and activity performance of children with DCD. Furthermore, the effects of MBI on fine motor skills and psychosocial factors in children with DCD require further validation. Additionally, it is unclear which delivery mode is more advantageous: group therapy or individualized sessions, the optimal intervention duration and frequency of MBI necessitate investigation, further research is warranted to delve into these matters. In addition, we would like to offer the following suggestions for future research. Firstly, all the studies included in our review were RCTs, which means that we may have overlooked some high-quality non-randomized controlled studies that could provide valuable insights. In future reviews, it will be beneficial to include studies that are properly assessed for risk of bias using appropriate tools, even if they are not randomized. Secondly, in future research designs, we recommend the use of controlled intervention designs (e.g., as seen in the Wood study [73]), ensuring that the only difference between the intervention and control groups throughout the training period is related to the primary variable of interest. We also emphasize the importance of providing a controlled intervention for the children in the control group, as engaging in some form of activity is always better than doing nothing, especially for children with DCD. Thirdly, the selection criteria applied across different studies represent a significant source of heterogeneity. To address this issue, future research should prioritize the use of standardized and consistent inclusion criteria, focusing on clinically diagnosed children with DCD, particularly those with more severe manifestations, and provide detailed information about the study sample. This approach will help minimize variability and enhance the comparability and reliability of study findings. Fourthly, The conclusions of this study are based on only a few existing studies involving clinical children with DCD. Although the results indicate that comprehensive rehabilitation training can significantly improve motor skills, body function, and activity performance, the number of studies and sample sizes are still limited. Therefore, future research should focus on increasing sample sizes and adopting stricter and more consistent inclusion criteria. Furthermore, there is a lack of comparative studies on different intervention methods, frequencies, or delivery modes in the current literature. Future studies should explore these aspects to provide more comprehensive intervention strategies for clinical practice. While existing research has confirmed that additional skill training helps improve motor skills, it has not yet clarified which intervention method is the most effective. Therefore, the next step in research should focus on exploring optimized intervention methods to identify the most effective approaches, especially those that best improve motor skills. Finally, the certainty of evidence in our study is rated as moderate or low, indicating that future research may alter the current findings. Therefore, we encourage researchers in this field to design more rigorous RCTs that comprehensively report their results. This should include the means and standard deviations for each group, details of the intervention environment, adverse effects, participant dropout rates, and long-term follow-up outcomes. Such studies would enable more robust meta-analyses and provide higher-level evidence to further determine the clinical significance of MBI.

## Conclusion

Our study provides substantial evidence supporting the conclusions of previous reviews, emphasizing that motor skill interventions, particularly task-oriented approaches, offer significant benefits for children with DCD. Specifically, our findings consistently demonstrate that motor skill interventions have a positive impact on a wide range of functional outcomes, including overall motor skills, balance, cognitive function, muscle function, coordination function, visual Function, sensory function, sensory organization function and activity performance. However, it is important to note that no statistically significant improvements were observed in psychosocial outcomes, indicating the need for further research in this area. In terms of participation outcomes, our study did not identify conclusive evidence supporting the effectiveness of MBI in improving participation abilities. This finding is based on the limited availability of data, with only one study reporting a negative outcome. This highlights the need for future investigations to examine this domain more comprehensively. Our study also provides valuable insights into the comparative effectiveness of different intervention strategies. Task-oriented approaches were shown to be particularly effective, yielding significant improvements in overall motor skills, balance, cognitive function, and activity performance. Similarly, combined task- and process-oriented strategies demonstrated notable improvements in overall motor skills, underscoring the potential benefits of integrated intervention approaches.

Nevertheless, due to the relatively small number of studies included in this meta-analysis, the effects of process-oriented strategies on overall motor skills and activity performance, as well as the effects of combined task- and process-oriented strategies on activity performance, remain inconclusive. Further high-quality studies are warranted to clarify these effects and to inform clinical practice. In conclusion, our findings suggest that task-oriented approaches and multi-level intervention strategies, such as combined task- and process-oriented methods, hold significant promise for addressing the diverse needs of children with DCD. These strategies should be flexibly applied within a multi-dimensional therapeutic framework to maximize functional outcomes.

Finally, we emphasize the urgent need for larger, methodologically rigorous RCTs to address the unresolved questions identified in our study. Future research should focus on: (1) Assessing the effects of MBI on participation levels and psychosocial outcomes in children with DCD; (2) Evaluating the impact of process-oriented strategies on overall motor skills and activity performance; (3) Investigating the effects of combined task- and process-oriented strategies on activity performance; (4) Provide detailed reports of the study sample and apply more stringent inclusion criteria. By addressing these gaps, future studies will help refine intervention approaches and ensure evidence-based clinical practices that better support children with DCD.

## Supplementary Information

Additional file 1.

## Data Availability

Data supporting the findings of this review are available from the corresponding authors.

## Abbreviations

DCD: Developmental coordination disorder
MBI: Motor-based interventions
ICD: International Classification of Diseases
ICF: International classification of functioning, disability and health
DSM: Diagnostic and Statistical Manual of Mental Disorders
MABC: Movement Assessment Battery for Children
BOT: Bruininks-Oseretsky Test of Motor Proficiency
TGMD: Test of Gross Motor Development
PRISMA: Preferred Reporting Items for Systematic Review and Meta-analysis
RCT: Randomized controlled trials
GRADE: Grading of Recommendation, Assessment, Development and Evaluation
