# Supplementary material for: Motor-Based Interventions in Children with Developmental Coordination Disorder: A Systematic Review and Meta-analysis of Randomised Controlled Trials
Source: Sports Med Open. 2025 May 26;11:59. doi: 10.1186/s40798-025-00833-w (PMC12106291; doi:10.1186/s40798-025-00833-w)
Supplement: Supplementary file 1 — Additional file 1. [file 40798_2025_833_MOESM1_ESM.docx]

**Motor-based interventions in children with developmental coordination disorder: A systematic review and meta-analysis of randomised controlled trials**

**Journal name：Sports Medicine-Open**

**Author:**

Jiaxin Gao^1，2†^, Yihan Yang ^3†^, Xiaqing Xu^2†^, Dunbing Huang^2^, Yangxin Wu^2^, Hongfei Ren^2^, Anren Zhang^2*^, Xiaohua Ke^2*^, Wei Song ^2*^

1 School of Health and Rehabilitation, Chengdu University of Traditional Chinese Medicine, Chengdu, China

2 Department of Rehabilitation Medicine, Shanghai Fourth People's Hospital, School of Medicine, Tongji University, Shanghai, China

3 College of Rehabilitation Medicine, Fujian University of Traditional Chinese Medicine, Fuzhou, China

† These authors contributed equally to this paper

* Correspondence should be addressed to Anren Zhang, Xiaohua [Ke and Wei Song; anren0124@tongji.edu.cn](mailto:Ke;anren0124@tongji.edu.cn), [kxh22@tongji.edu.cn](mailto:kxh22@tongji.edu.cn), [songwei94@tongji.edu.cn](mailto:songwei94@tongji.edu.cn) .

**Online Resource 1 . Search terms**

The following terms were used as text words and key words: (Motor Skills Disorders OR Motor Skills Disorders OR Developmental Coordination Disorder OR Coordination Disorder, Developmental OR Developmental Coordination Disorders AND (therapy OR treatment OR intervention OR training OR rehabilitation) AND (Child OR Children OR childhood OR youth OR adolescent OR adolescence).
